# Comparative and phylogenetic analyses based on the complete chloroplast genome of *Cornus* subg. *Syncarpea* (Cornaceae) species

**DOI:** 10.3389/fpls.2024.1306196

**Published:** 2024-03-13

**Authors:** Bicai Guan, Jianteng Wen, Hanjing Guo, Yizhen Liu

**Affiliations:** College of Life Sciences, Nanchang University, Nanchang, China

**Keywords:** *Cornus* subg. *Syncarpea*, classification, chloroplast genome, phylogenetic analysis, comparative analysis

## Abstract

This study presents a comprehensive analysis of the chloroplast (cp) genomes of *Cornus* species, including comparative and phylogenetic evaluations, as well as examinations of their genomic structure and composition. The cp genomes exhibit a typical circular quadripartite structure and demonstrate highly similar gene order and genomic structure. The complete cp genome size of the 10 taxa in this study is 156,965 bp to 157,383 bp, where the length of the large single-copy (LSC) region is 86,296 bp to 86,691 bp, small single-copy (SSC) region is 18,386 bp to 18,454 bp, and inverted repeat (IR) region is 23,143 bp to 26,112 bp. A total of 131 genes were found, including 86 protein-coding genes (PCGs), eight rRNA genes, and 37 tRNA genes. The mean GC content of the 10 taxa is 38.145%, where the LSC region is 36.396%, the SSC region is 32.372%, and the IR region is 43.076%. Despite the relatively conserved nature of the cp genome within the species of *Cornus*, 25–31 simple sequence repeats (SSRs) were identified in the 10 taxa in our study. The SSRs were found to be distributed in the LSC, SSC, and IR regions in *Cornus hongkongensis* subsp. *hongkongensis*, *C. hongkongensis* subsp. *elegans*, *C. hongkongensis* subsp. *gigantea*, and *C. hongkongensis* subsp. *tonkinensis*, while the SSR was not found in the IR region of the other six taxa. Thus, whole cp genomics is a valuable tool for species identification, taxonomic clarification, and genomic evolutionary analysis. Furthermore, our findings reveal that *C. hongkongensis* and *C. hongkongensis* subsp. *gigantea*, along with *Cornus kousa* and *Cornus elliptica*, form sister groups. Notably, *C. hongkongensis* subsp. *ferruginea* and *C. hongkongensis* subsp. *melanotricha* did not exhibit affinity with *C. hongkongensis* subsp. *hongkongensis*. Our study furnishes essential data for further research on their classification and provides novel insights into the relationship within *Cornus* subg. *Syncarpea*.

## 1 Introduction

The classification of the species within the *Cornus* genus has been a subject of controversy, yielding varying conclusions drawn from different studies. As early as the 19th century, Lindley (1883) separated the *Cornus elliptica* Wall. from *Swida* and named it “*Benthamia*” (Xiang, 1987), and several different classifications have emerged over time. In Flora Reipublicae Popularis Sinicae, there are 10 species included in the *Dendrobenthamia* genus. However, in the Flora of China, this genus has been downgraded to a subgenus (*Cornus* subg. *Syncarpea*), with some species having been merged, resulting in only five remaining species (Xiang and David, 2005). Since the 1950s, more than 15 new taxa have been described. In a treatment following Xiang’s (1987) classification, five species, namely, *Cornus capitata*, *Cornus hongkongensis*, *Cornus kousa*, *Cornus multinervosa*, and *C. elliptica*, were recognized with 13 subspecies. However, a preliminary allozyme investigation by Dudley and Santamour revealed a significant divergence between *C. capitata* subsp. *capitata* and *C. capitata* subsp. *angustata*, prompting the recognition of *C. elliptica* as a distinct species. Further research is necessary to clarify the classification of the various species within this group. Advances in high-throughput sequencing technologies have enabled the use of cp genomes for phylogenetic studies.

The morphological characteristics of plants can vary due to external factors such as soil, light, precipitation, temperature, and natural enemies. This implies that plant species identification based solely on phenotype characteristics is challenging and even controversial in terms of its validity. Apart from extrinsic influencing factors, plants’ own genes also undergo changes during adaptation to the environment or climate change. In this regard, the cp genome offers several advantages: 1) cp DNA has a higher copy number within the overall cell DNA and is easily obtainable through sequencing; 2) cp DNA is primarily maternally inherited with a conservative gene structure and minimal gene reorganization; 3) the long sequence of cp genes provides sufficient genetic information for resolving issues related to species classification and evolution.

It is widely acknowledged that the process of photosynthesis is crucial for the energy and biomass production of plants, with chloroplasts playing a pivotal role (Wu et al., 2016; Tang et al., 2018). Chloroplasts are self-replicating organelles composed of homogeneous circular DNA molecules. The genetic information contained within their genomes is maternally inherited from generation to generation (Birky, 1995). The double-stranded DNA in the cp genome ranges from 70 to 520 kb in algae, while it is typically more conserved in land plants, with a range of 120 to 160 kb (Song et al., 2022; Osuna-Mascaró et al., 2018). The cp genomes of land plants are generally stable and typically contain four highly conserved regions: a small single-copy (SSC), a large single-copy (LSC), and a pair of inverted repeat (IR) regions (IRa and IRb) (Mower and Vickrey, 2018). Despite their conservative nature, there are significant variations in genome sizes and gene types among them, such as insertions, substitutions, and missing nucleotide sites. Additionally, expansions and contractions of IR regions as well as translocations and rearrangements of genes have been observed (Ahmed et al., 2012; Sloan et al., 2013). This diversity and polymorphism can be applied to phylogenetic analysis, population taxonomic and genetic research, and evolutionary investigations (Lössl and Waheed, 2011; Ahmed, 2015). Furthermore, cp genomes contain a wealth of phylogenetic information with a mutation rate sufficient for phylogenetic inference and species partitioning (Moore et al., 2010). In addition, plant cp genomes are characterized by a conserved structure and a high substitution rate, rendering them a valuable source for plant molecular identification, genetic diversity assessment, and phylogenetic analysis (Dong et al., 2012, 2014). The advent of next-generation sequencing technology has provided an efficient and cost-effective method for cp genome assembly, greatly enriching cp genome information while enabling the application of cp genomes to lower taxonomic levels in order to resolve the classification relationship among related species (Dong et al., 2012) and providing sufficient data for plant phylogenetic studies (Cronn et al., 2008; Tangphatsornruang et al., 2010). Nonetheless, there is a paucity of reports about the cp genome of *Cornus* subg. *Syncarpea*.

Regrettably, reports on the cp genome of *Cornus* subg. *Syncarpea* are scarce, hampering its potential for genetic information discovery and phylogenetic studies. The entirety of cp genomes furnishes more comprehensive genetic information compared to mere single gene fragments, which facilitates a better understanding of inter-specific genetic resources and evolutionary history. Nevertheless, prior research on *Cornus* subg. *Syncarpea* has inadequately scrutinized the inter-species relationship and genomics, impeding a comprehensive comprehension of its phylogeny. Conversely, inter-species relationship research of other species has been scrutinized through cp genomics (Song et al., 2019), yielding a reference for the inter-specific relationship of *Cornus* subg. *Syncarpea*. Therefore, this study endeavors to employ comparative cp genomics to address the ensuing issues: 1) to comprehensively comprehend the cp genome genetics of *Cornus* subg. *Syncarpea*; 2) to deduce that differences in simple sequence repeats between species, including their number and location, can furnish a molecular-level reference for subsequent exploration of *Cornus* subg. *Syncarpea*; and 3) to clarify the relatedness of the species within *Cornus* subg. *Syncarpea*, providing a scientific foundation for the classification at the species order level.

## 2 Materials and methods

### 2.1 Sampling, extraction, and genome sequencing

Ten specimens belonging to *Cornus* subg. *Syncarpea* were collected from diverse locations across China (S1). The fresh leaves were cleaned to remove stains and impurities from the surface of the leaves, excess water was removed, and the leaves were put into the silicone to dry thoroughly so that the treated leaves could be preserved for a long time, as recommended by Chase and Hills (2019). The complete cp genomic DNA of 10 samples belonging to *Cornus* subg. *Syncarpea* was extracted using a modified cetyl trimethylammonium bromide (CTAB) method. To obtain the cp genome, Huitong Biotechnology’s approach was employed to address challenges associated with extracting organellar DNA from total DNA. Initially, total cellular DNA was extracted and subsequently sequenced. Following sequence assembly, organelle DNA contigs were screened based on similarity and close sequence matches (Wang et al., 2019). The overall method involved selecting higher-quality sequences that matched well while considering their characteristics for further screening and splicing. PCR amplification products were used to fill gaps between contigs based on the annotation locations obtained after splicing, thus completing the entire organelle genome (Deng et al., 2017; Elpe et al., 2018). Additionally, the higher coverage of cp genomes in sequencing data compared to nuclear and mitochondrial genomes serves as an important criterion for distinguishing them. Sequencing was performed at Huitong Biotechnology Co., Ltd. (Shenzhen, China) utilizing the Illumina paired-end technology platform. After qualifying the samples through agarose gel electrophoresis analysis, the purified high-quality genomic DNA was randomly fragmented using the Covaris Ultrasonic Disintegrator. Subsequently, a series of treatments including end repair, A-tailing, sequencing adapter addition, purification, and PCR amplification were performed to construct sequencing adapter paired-end (PE) libraries (Jing et al., 2022). Following library construction, preliminarily quantification of the library was conducted using Qubit2.0 after dilution while detecting insert fragments using Agilent 2100 for size confirmation. Once the size criteria for insert fragments were met, accurate quantification of effective library concentration was determined using the Q-PCR method to ensure library quality control measures were in place prior to sequencing on an Illumina NovaSeq6000 platform in 150-bp paired-end mode (Tang et al., 2022).

### 2.2 Assembly and annotation of chloroplast genome

Low-quality data were filtered for raw data using NGS QC Tools Kit software, including reads with more than 5% N bases, low quality (mass ≤5), 50% base number, and reads with adapter contamination, resulting in clean reads. Then, SPAdes (version 3.11.0) was employed to assemble the clean reads, utilizing default parameters while disregarding the cut-off parameter (Ma et al., 2020). All identified scaffolds were collated from the clean data. To ensure accuracy, blastn and Exonerate analyses were conducted using published closed-source cp data and protein-coding gene sequences as reference material, respectively. The threshold was set to evalue 1e^−10^ for blastn and a protein similarity threshold of 70% for Exonerate. Scaffolds with matching genes were selected and sorted by splicing coverage, discarding any segments that were evidently not part of the target genome. Low-coverage segments were also excluded, with most being at 1,000× and only a few at 10× (Li et al., 2020). The collected fragmented target sequences were then extended and merged using PRICE and MITObim to minimize the number of scaffolds, with 50 iterations being the standard (Chen et al., 2020). For iterative splicing results, bowtie2 was utilized to match the original sequencing reads (Jin et al., 2020), and the matched paired reads were selected and re-spliced using SPAdes software. The path was scrutinized to identify the presence of an obvious ring map, extracting it if so. If not, steps 3–5 were repeated until the circular genome was complete (Xia and Liu, 2020; Zhou et al., 2020).

Both the initial and re-splicing processes employed SPAdes software. The default kmer setting was utilized for the initial splicing, while the –careful mode was used for self-correction of the hammer and reply of post-splicing to correct the sequence. The results were optimized during the re-splicing process, utilizing VelvetOptimiser to optimize the kmer settings. The subsequent repeated re-splicing process utilized kmer values of 93, 95, 97, 103, 105, 107, and 115, which were then spliced and integrated (Shao and Jiang, 2020; Zhao and Gao, 2020), the graph file was obtained, the graph file was visualized by Bandage software, and the excess contig was removed and edited into a circular sequence, the complete cp complete genome sequence.

Upon completion of homology and protein sequence alignments, the organelle sequences were consolidated into separate fasta files. The iterative extension was then performed utilizing PRICE (Paired-Read Iterative Contig Extension) until a stable and consistent sequence length was attained. PRICE harnesses paired-end sequencing data to match contig ends, allowing for the iterative lengthening of contigs and the merging of overlapping ones. Subsequent sequence recovery was carried out through the following command: “PriceTI -fpp 1.fq 2.fq 600 95 -nc 50 -dbmax 72 -mol 30 -mpi 90 -target 90 2 1 1 -o extension.fasta -icf contigs.fasta 1 1 5”. Here, an insert length of 300 bp was set (double-ended 150-bp read length plus a 300-bp insert fragment, with an estimated total length of 600 bp). The reply reads were required to have a minimum similarity of 95% with the contig. Iterative assembly was performed using kmer, with a maximum read length of 72 bp (-dbmax 72). Overlapping reads and contig edges had a minimum overlap length of 30 bp (-mol 30). The matching reads had at least 90% uniformity (-mpi 90), with the extension mode enabled (-target). After each extension, bowtie2 (-N 1 parameter) was utilized to map reads, and reads that were consistent with the extended sequence were extracted as input for subsequent reassembly. The cp genome was annotated using Plastid Genome Annotator (PGA) and to ensure the accuracy of the annotation results, the genome was also annotated simultaneously with the help of GeSeq online tool2 (Tillich et al., 2017). To validate annotated genes, Geneious-v10.2.3 software (Kearse et al., 2012) was utilized to further refine and manually correct errors in gene annotation. Particular attention was given to genes located at boundaries and highly variable genes, such as *ndhF*, *ndhK*, *ycf2*, and *accD*. Finally, the circular cp genome map of *Cornus* subg. *Syncarpea* was created and visualized using the OGDraw online tool^3^ (Greiner et al., 2019).

### 2.3 Comparative analysis of the chloroplast genome

To assess the cp genome characteristics of 10 taxa of *Cornus* subg. *Syncarpea*, Geneious-v10.2.3 software was utilized. To compare the cp genomes of these 10 taxa with 12 out-groups, genomic similarity analysis was performed using the Glocal alignment program (shuffle-LAGAN mode) in m-VISTA (Brudno et al., 2003; Frazer et al., 2004). Moreover, a boundary analysis of SC/IR was carried out using IRscope (Amiryousefi et al., 2018) to observe any expansions or contractions of genes at the borders. Codon usage bias analysis was performed using MEGA 7.0 software (Kumar et al., 2016).

### 2.4 Codon usage analysis

Relative synonymous codon usage (RSCU) is a measure of the relationship between observed and expected frequencies of codons in the case of random usage of preferred synonymous codons. An RSCU value greater than 1.6 indicates an overrepresented codon, while an RSCU value less than 0.6 indicates an underrepresented codon (Behura and Severson, 2012). RSCU can be calculated using the following formula:


$$RSCU_{\text{ij}}=\frac{X_{\text{ij}}}{\frac{1}{\text{n}}\sum_{\text{j}-1}^{\text{ij}}X_{\text{ij}}}$$

where X_ij_ is the observed number of the ith codon for the jth amino acid, ni is the total number of synonymous codons that encode the jth amino acid, and fi is the frequency of the ith codon among all synonymous codons for the jth amino acid.

To analyze codon usage and amino acid frequency, Geneious Prime 2020 was utilized (Kearse et al., 2012), while RSCU of protein-coding genes was evaluated using MEGA-X (Kumar et al., 2018).

### 2.5 Analysis of repeats

The molecular marker value of simple sequence repeats (SSRs) in biological research is highly significant. SSR represents a DNA molecular marker technology, with its core being PCR technology (Bi et al., 2020). Consequently, the advancement of simple repetitive sequences plays a crucial role in subsequent relevant molecular marker techniques. Simple repetitive sequences belong to the second generation of genetic markers and find applications in various fields such as genetic mapping, gene mapping, forensic identification, anthropology, and genetic disease diagnosis (Yan et al., 2020).

To identify repeats, the REPuter tool^4^ was employed, which is capable of detecting forward, reverse, palindrome, and complement sequences. The Hamming distance was set to 3, and the minimum length and identity of repeats were restricted to ≥30 bp and >90%, respectively. The SSRs were analyzed using MISA (Beier et al., 2017), with a basic repeat setting and a threshold of 10, 8, 4, 4, 3, and 3 for mono-, di-, tri-, tetra-, penta-, and hexanucleotides, respectively.

### 2.6 Phylogenetic analysis

To elucidate inter-species relationships among 10 taxa of *Cornus* subg. *Syncarpea*, a phylogenetic tree was constructed using maximum parsimony (MP) and Bayesian inference (BI) methods. Ten species and subspecies from *Cornus* subg. *Syncarpea* were included, while 12 species (S2), such as *Cornus florida*, were selected as out-groups. Phylogenetic analyses were conducted using five different data sets, each analyzed separately. These comprised 22 cp whole-genome data, 10 cp gene coding sequence (CDS region) data, 10 cp gene LSC region data, 10 cp gene intron data, and 10 cp gene IR region data. The first data set included 22 cp whole-genome data from 10 taxa of *Cornus* subg. *Syncarpea* and 12 out-groups, which had already been annotated. Each data set was initially aligned using the software MAFFT-v7.409, based on default parameters (Katoh and Standley, 2013). Stop codons were removed, and bad fragments were abandoned using the Gblock program (Talavera and Castresana, 2007). Finally, an MP tree was constructed using MEGA X (Bi et al., 2020). Phylogenetic tree inference by the Bayesian methods was performed using Mrbayes 3.2.6. Two hot chains and two cold chains were set to run 2,000,000 generations for inspection, node BI values were converged and stabilized, every 1,000 generations were counted, the top 25% of trees were discarded as aging trees, and the rest were discussed to infer tree structure. The phylogenetic trees were visualized using the software Figtree-v1.4.45, and the results were imported into the software Figtree-v1.4.4 to enhance their visualization.

## 3 Results

### 3.1 Chloroplast genome features

**Figure 1:**
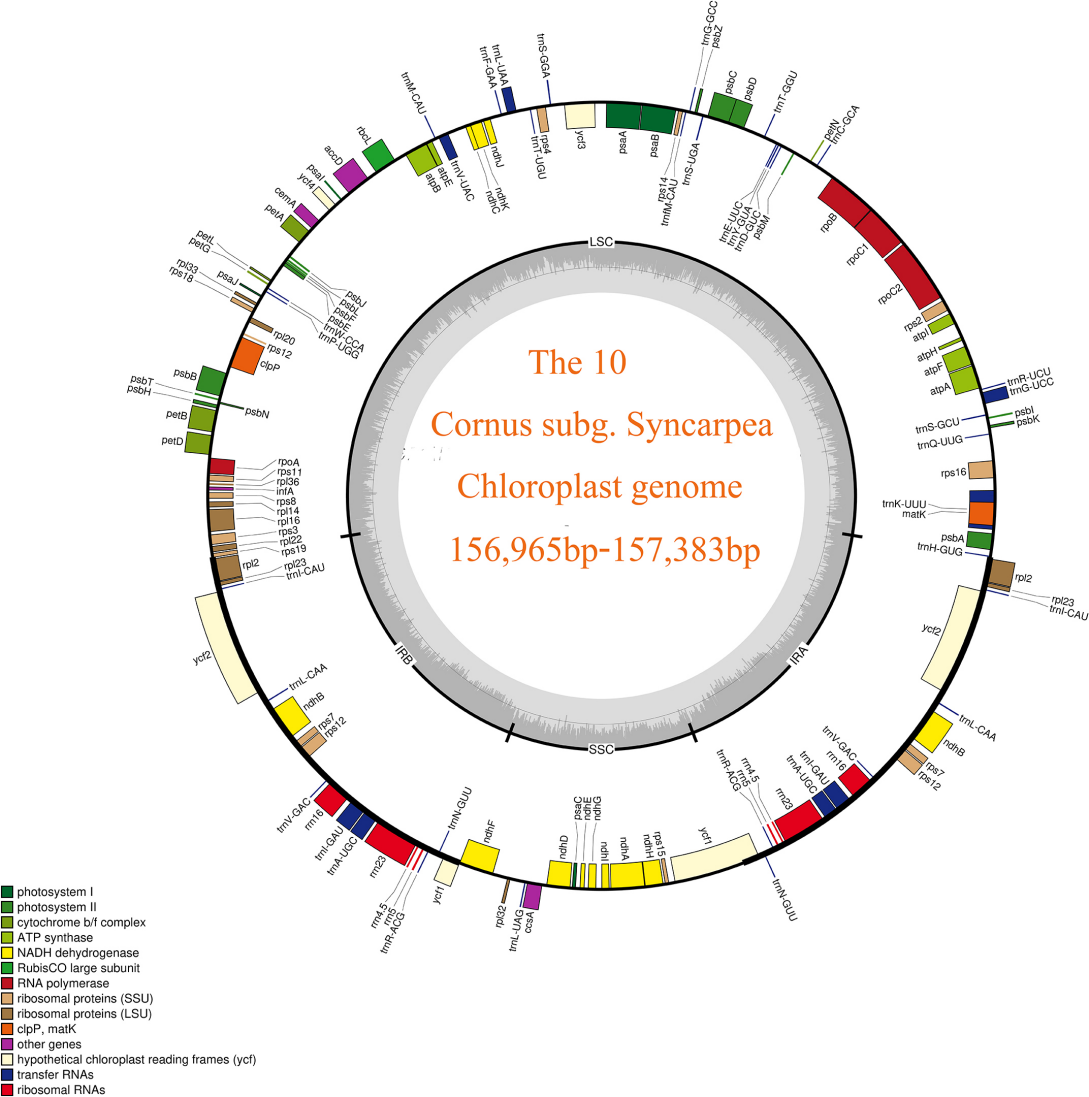
Chloroplast genome map of 10 taxa of *Cornus* subg. *Syncarpea*. The transcription direction of the genes in the outer circle in the figure is counterclockwise, and the transcription direction of the genes in the inner circle is clockwise. Different colors indicate that the function of the coding gene is also different. The gray part of the inner circle indicates the GC content, which is distinguished by depth.

A staggering 40.65 G of raw data was generated from the genomic libraries, which was then refined to obtain 40.44 G of clean data, and the final number of raw reads of 10 taxa ranged from 12,523,414 to 14,894,202, while the number of clean reads after filtering ranged from 12,451,564 to 14,810,440 (S2). Using the visualization tool OGdraw, 10 chloroplast genome maps of the *Cornus* subg. *Syncarpea* were obtained (**Figure 1**). The assembly of the complete cp genome for 10 taxa of *Cornus* subg. *Syncarpea* yielded a genome size that ranged from 156,965 bp to 157,383 bp, inclusive of four regions that are ubiquitous in land plants and form a loop structure. These four regions comprised an LSC region spanning from 86,296 bp to 86,691 bp, an SSC region ranging from 18,386 bp to 18,454 bp, and two IR regions spanning from 23,143 bp to 26,112 bp (**Table 1**). In addition, the two single-copy regions were demarcated by a pair of IR regions. Across all 10 taxa of *Cornus* subg. *Syncarpea*, the GC content of the cp genome ranged from 38.12% to 38.17%, and the GC content of the IR regions (ranging from 43.06% to 43.1%) exceeded that of the LSC (ranging from 36.36% to 36.43%) and SSC (ranging from 32.35% to 32.4%) regions. It is worth noting that *C. capitata* exhibited the highest GC content of 43.36% in its IR regions. The lowest sequencing depth value was 180× of the *C. hongkongensis* subsp. *gigantea*, and the largest sequencing depth value was 1,055× of the *C. hongkongensis* subsp. *ferruginea*, so the error rate of the sequencing results was very low, and the sequencing results were highly credible (**Table 1**).

**Table 1:** Features of the chloroplast genomes of 10 plants of *Cornus* subg. *Syncarpea*.

Species	Genome size (bp)				GC content (%)				Genes				Depth
	Total	LSC	SSC	IR	Total	LSC	SSC	IR	Total	PCGs	rRNA	tRNA	
*Cornus hongkongensis* subsp. *gigantea*	156,980	86,314	18,394	26,136	38.17	36.42	32.38	43.08	131	86	8	37	180
*Cornus hongkongensis* subsp. *hongkongensis*	156,989	86,327	18,392	26,137	38.16	36.42	32.38	43.08	131	86	8	37	309
*Cornus multinervosa*	157,365	86,621	18,454	26,143	38.12	36.36	32.35	43.07	131	86	8	37	271
*Cornus hongkongensis* subsp. *elegans*	157,197	86,537	18,386	26,137	38.14	36.39	32.37	43.07	131	86	8	37	392
*Cornus hongkongensis* subsp. *tonkinensis*	156,965	86,296	18,395	26,137	38.17	36.42	32.39	43.08	131	86	8	37	436
*Cornus capitata*	157,199	86,563	18,412	26,112	38.17	36.43	32.40	43.10	131	86	8	37	848
*Cornus hongkongensis* subsp. *melanotricha*	157,288	86,620	18,424	26,122	38.15	36.39	32.40	43.08	131	86	8	37	474
*Cornus hongkongensis* subsp. *ferruginea*	157,291	86,605	18,432	26,127	38.13	36.39	32.35	43.07	131	86	8	37	1,055
*Cornus kousa*	157,380	86,689	18,437	26,127	38.12	36.37	32.35	43.07	131	86	8	37	553
*Cornus elliptica*	157,383	86,691	18,438	26,127	38.12	36.37	32.35	43.06	131	86	8	37	411

LSC, large single copy; SSC, small single copy; IR, inverted repeat; PCGs, protein-coding genes.

A total of 131 genes were meticulously annotated in the cp genomes of the 10 *Cornus* subg. *Syncarpea* species and subspecies, encompassing 86 protein-coding genes (PCGs), eight rRNA genes, and 37 tRNA genes. These genes were methodically grouped into three categories based on their specific functions, while 15 genes including *trnK-UUU*, *rps16*, *trnG-UCC*, *atpF*, *rpoC1*, *trnL-UAA*, *trnV-UAC*, *petB*, *petD*, *rpl16*, *rpl2*, *ndhB*, *trnI-GAU*, *trnA-UGC*, and *ndhA* featured one intron; *clpP* and *ycf3* genes exhibited two introns; and *rps12* gene underwent trans-splicing (see **Table 2** for details).

**Table 2:** List of the annotated genes in the chloroplast genomes of *Cornus* subg. *Syncarpea*.

^Category^	Groups of gene	Name of genes
Self-replication	Ribosomal RNA Transfer RNA	*rrn4.5, rrn5, rrn16, rrn23* *trnA-UGC^a,c^, trnC-GCA, trnD-GUC*, *trnE-UUC, trnF-GAA, trnfM-CAU*, *trnG-GCC, trnG-UCCa,trnH-GUG^c^*, *trnI-CAUc, trnI-GAU^a,c^, trnK-UUU^a^*, *trnL-CAAc, trnL-UAA^a^, trnL-UAG, trnM-CAU, trnN-GUUc, trnP-UGG, trnQ-UUG, trnR-UCU, trnR-ACG^c^, trnS-UGA, trnS-GCU, trnS-GGA, trnT-GGU, trnT-UGU, trnV-UAC^a^*, *trnV-GACc, trnW-CCA,trnY-GUA*
	Small subunit of ribosome	*rps2, rps3, rps4, rps7^c^, rps8, rps11*, *rps12^a,c^, rps14, rps15^c^, rps16^a^*, *rps18, rps19^c^*
Photosystem I	Large sub unit of ribosome	*rpl2^a,c^, rpl14, rpl16^a^, rpl20*, *rpl22, rpl23^c^, rpl32, rpl33, rpl36*
	RNA polymerase subunits	*rpoA, rpoB, rpoC1, rpoC2 Photosynthesis* *psaA, psaB, psaC, psaI, psaJ*, *cf1, ycf2, ycf3b, ycf4*
	Photosystem II	*psbA, psbB, psbC, psbD, psbE*, *psbH, psbI, psbJ, psbK, psbL*, *psbM, psbF, psbN, psbT, psbZ*
	Sub units of cytochrome ATP synthase NADH-dehydrogenase	*petA, petB^a^, petD^a^, petG, petL, petN* *atpA, atpB, atpE, atpF^a^, atpH, atpI* *ndhA^a^, ndhB^a,c^, ndhC, ndhD, ndhE*, *ndhF, ndhG, ndhH, ndhI, ndhJ, ndhK*
Othergenes	Rubiscolargesubunit Translational initiation factor Maturase K Envelope membrane protein Proteolysis Cytochrome c biogenesis acetyl-CoAcarboxylase beta subunit	rbcL infA matK cemA clpP clpP ccsA accD

a Genes with one intron.

b Genes with two introns.

c Two genes copied in IR regions.

### 3.2 Junction characteristics

**Figure 2:**
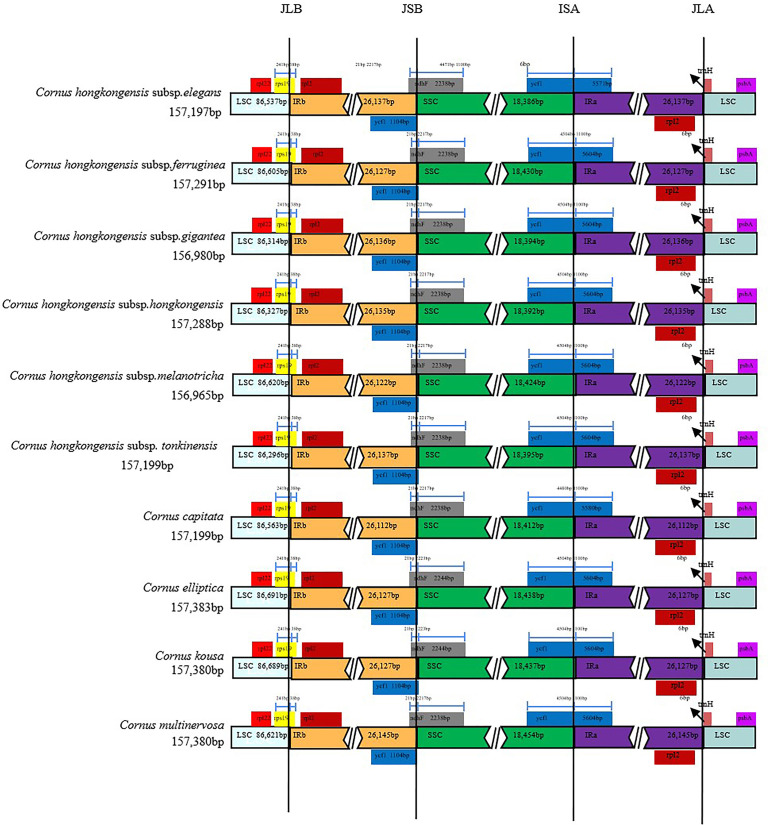
Alignment of SC/IR boundary of chloroplast genomes of 10 taxa of *Cornus* subg. *Syncarpea*. The different colors in the figure indicate that the function of the coding gene is also different. The number above the gene indicates the distance between the end of the gene and the boundary position.

We conducted a comparative analysis of junction structures in the cp genomes of 10 taxa of *Cornus* subg. *Syncarpea* to observe variations in their IR boundaries (**Figure 2**). Our results revealed striking similarities in their boundary features, with genes at the nodes primarily consisting of *rpl22*, *rps19*, *rpl2*, *ycf1*, *ndhF*, *trnH*, and *psbA*. Specifically, the *rpl2* and *rpl22* genes were replicated and fully embedded in the IRb and LSC regions, respectively. The *trnH* genes were entirely located to the left of the IRa/LSC junction, 6 bp from this boundary. The *rps19* genes occupied the IRb/LSC junction, with only a small portion of 38 bp extending into the IRb region. The *ndhF* genes occupied the IRb/SSC junction, with only a small portion of 21 bp extending into the IRb region. Intriguingly, we discovered that the *ycf1* genes were fully embedded in the IRb region when located in the IRb/SSC junction while occupying the IRa/SSC junction and mainly located within the SSC region. Furthermore, we observed differences between *C. hongkongensis* subsp. *elegans* and *C. capitata*, with 1,100 bp located within the IRa region for all 10 taxa of *Cornus* subg. *Syncarpea* and 4,480 bp of *C. capitata* extending into the SSC region, compared to 4,471 bp of *C. hongkongensis* subsp. *elegans*.

### 3.3 Similarity analysis of chloroplast genomes

**Figure 3:**
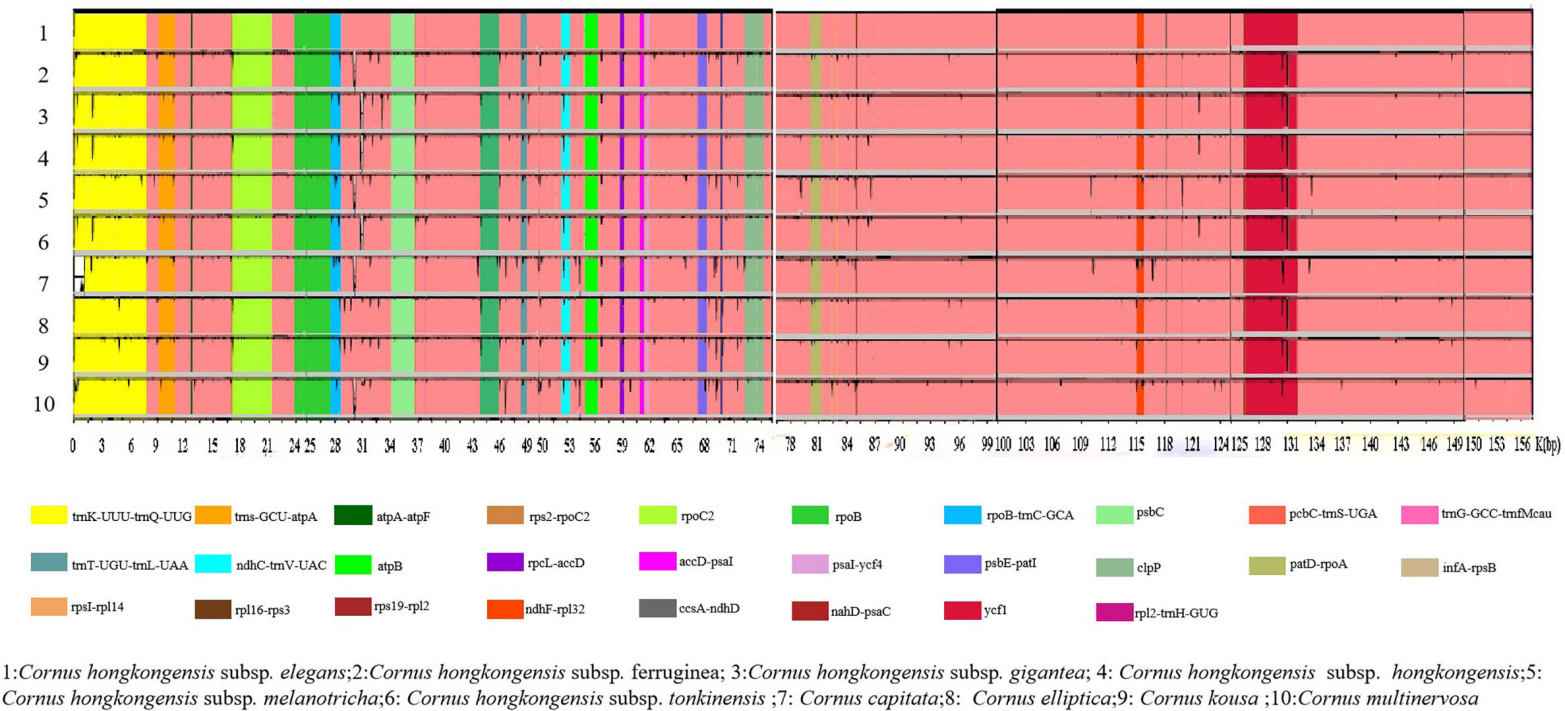
Whole sequence alignment of the chloroplast genomes of 10 taxa of *Cornus* subg. *Syncarpea*.

Utilizing m-VISTA and DnaSP, a comparative analysis of the complete cp genomes of 10 taxa belonging to the *Cornus* subg. *Syncarpea* was conducted. This investigation aimed to detect hyper-variable regions, construct sequence identity plots, and identify structural differences (**Figure 3**). Though the number and arrangement of genes were primarily identical across these species, two differential genes (*ndhF* and *ycf1*) were observed in the SSC region. Overall, non-coding regions exhibited a greater potential for variation than coding regions. While the protein-coding regions were relatively conserved, larger variants were detected in the *rpoB-trnC-GCA* and *ndhC-trnV-UAC* genes. However, the ATP synthase and Photosystem I displayed a high degree of conservation, with genes such as *atpA-atpF*, *atpB*, and *psaI-ycf4* remaining virtually unaltered. Moreover, the IR regions of these species remained mostly unaltered and were notably more conserved than the two single-copy regions.

### 3.4 Codon usage analysis

**Figure 4:**
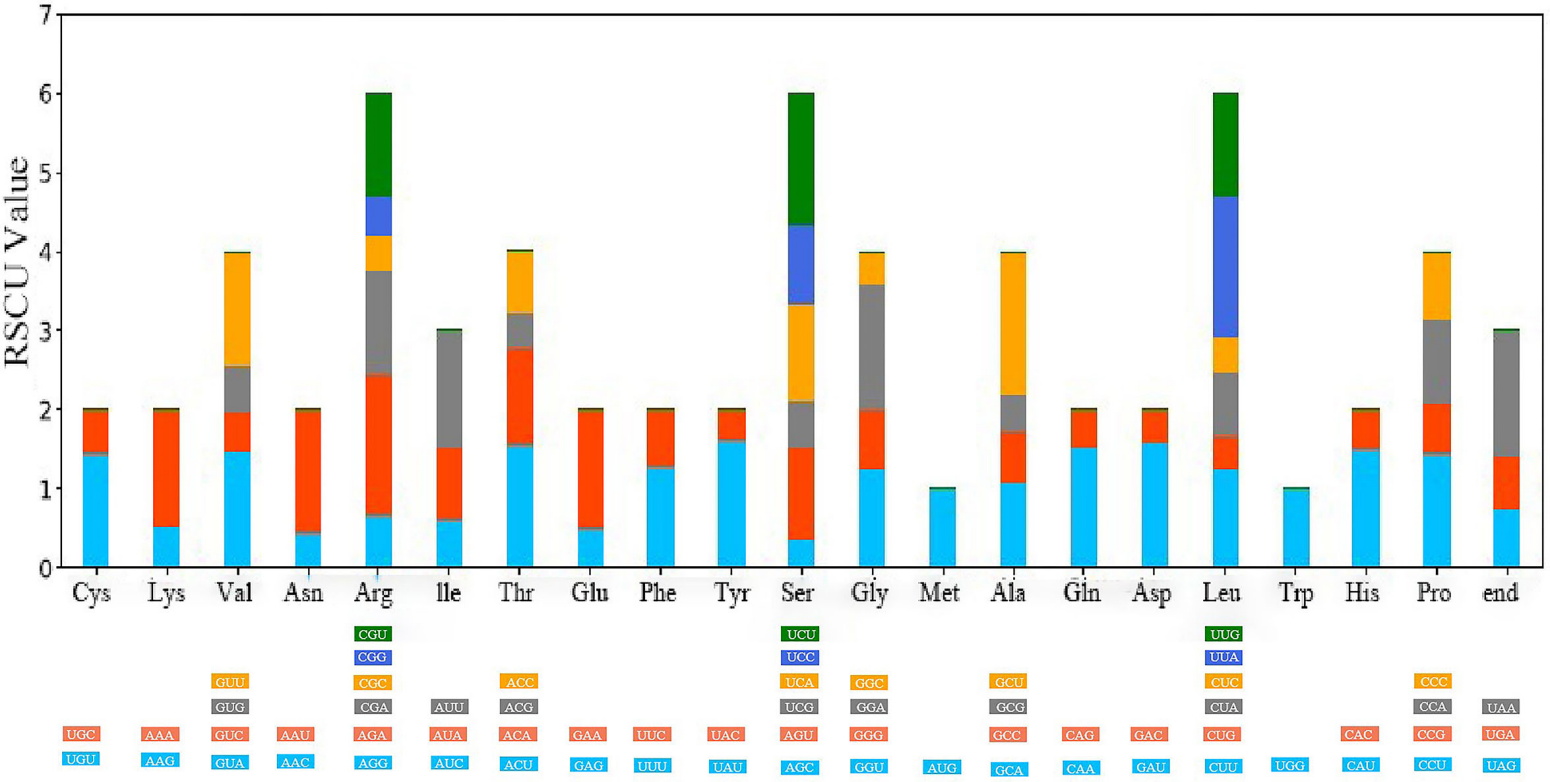
Relative synonymous codon usage (RSCU) values for amino acids and stop codons of *Cornus capitata*. The colors of the histograms correspond to the colors of the codons.

**Figure 5:**
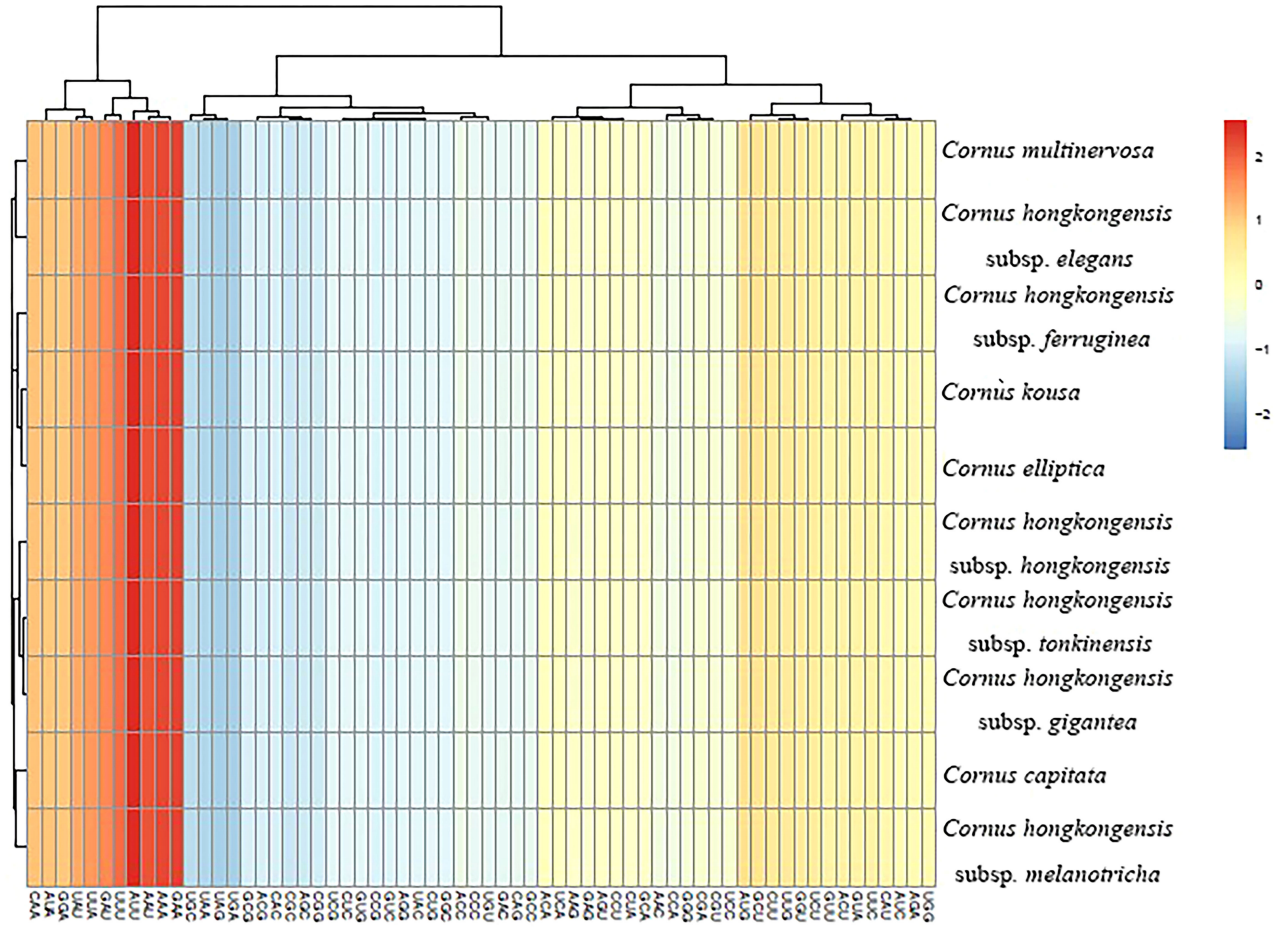
Heat map of codon usage frequency.

Upon analyzing the codon usage in 10 taxa belonging to the *Cornus* subg. *Syncarpea*, it was discovered that methionine and tryptophan amino acids were coded by solitary codons, AUG and UGG, respectively. However, the remaining amino acids were coded by two to six codons, with a clear preference for certain codons (**Figure 4**). Notably, among all codons encoding amino acids, AUU was the most commonly utilized (**Figure 5**). Interestingly, the majority of codons with RSCU values greater than 1 had A/U as the terminal codon, while those with C/G as the terminal codon typically had RSCU values less than 1.

### 3.5 Nucleotide repeat analysis

**Figure 6:**
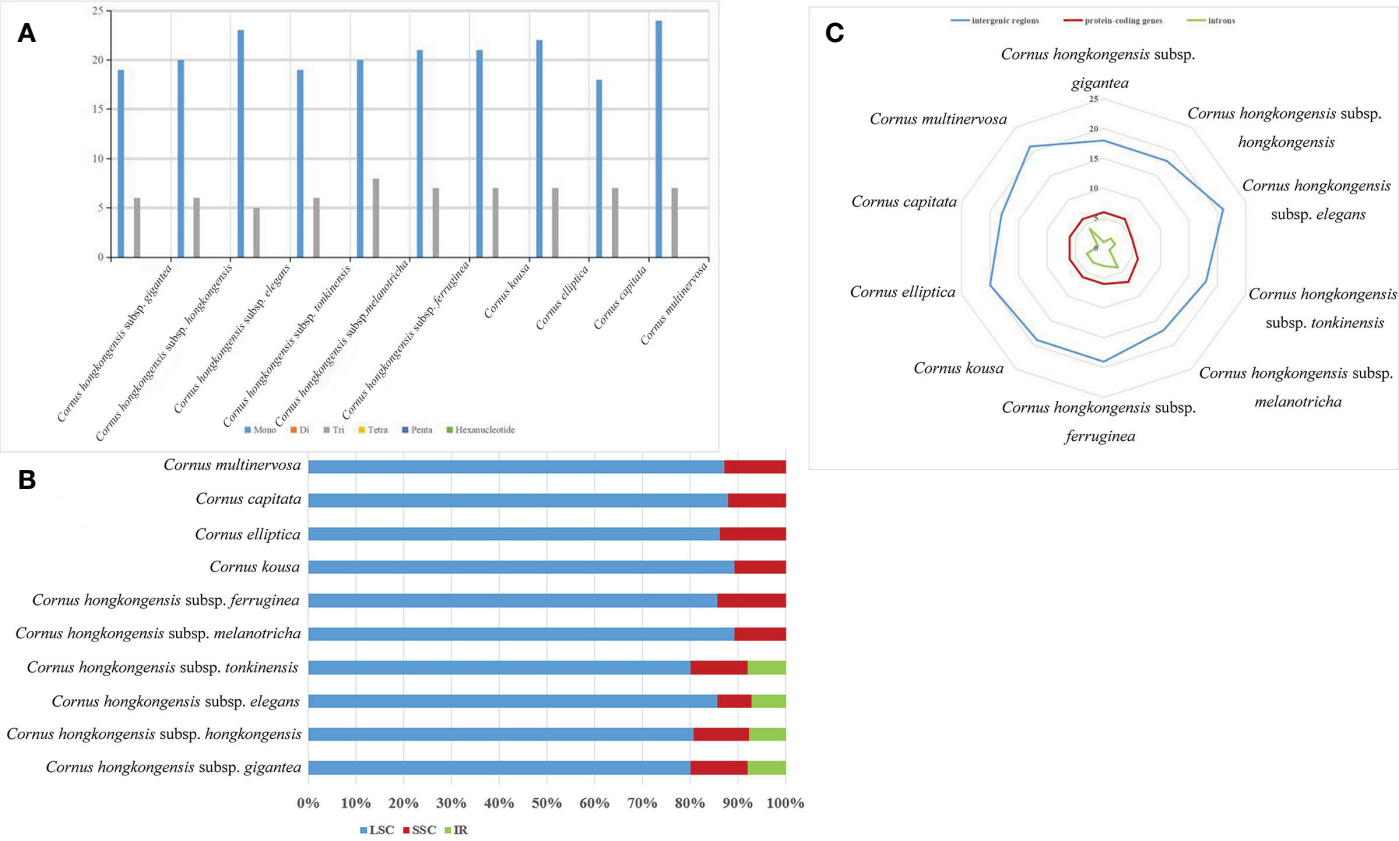
Comparison of SSR analysis of the chloroplast genomes of 10 taxa of *Cornus* subg. *Syncarpea.* **(A)** The number of different SSR types. **(B)** The frequency of SSR occurrence in the LSC, IR, and SSC regions. **(C)** The frequency of SSRs in intergenic regions, protein-coding genes, and introns. SSR, simple sequence repeat; LSC, large single copy; IR, inverted repeat; SSC, small single copy.

SSRs, which are short (1–6 bp) tandemly repeated DNA sequences present in the genome, are widely used as molecular markers and play a crucial role in plant identification and classification (Luo et al., 2021). Nucleotide repeats are dominated by mononucleotide repeats, whereas dinucleotides, trinucleotides, and even hexanucleotides appear less repeatedly. In our study, mononucleotide and trinucleotide repeats were the only repeats detected in the cp genomes of 10 taxa belonging to the *Cornus* subg. *Syncarpea*, with mononucleotide repeats being more abundant than trinucleotide repeats (**Figure 6A**), which is similar to the chloroplast genome studies in other plants (Chen et al., 2023; Fu et al., 2023). The SSRs were primarily distributed in the LSC region, accounting for over 80% of the total distribution. The SSC regions followed with a distribution of less than 15%. The lowest occurrence of SSRs was observed in the IR regions, comprising less than 10%. Furthermore, no SSR distribution was detected in the IR region of six taxa: *C. multinervosa*, *C. capitata*, *C. hongkongensis* subsp. *melanotricha*, *C. hongkongensis* subsp. *ferruginea*, *C. kousa*, and *C. elliptica* (**Figure 6B**). The frequency distribution of these SSRs ranged from 17 to 21 repeats in gene spacers, five to seven repeats in protein-coding genes, and one to four repeats in introns. Notably, there were fewer variations of SSRs observed in protein-coding regions compared to spacer and intron regions (**Figure 6C**). A total of 25–31 SSRs were detected in the 10 taxa of *Cornus* subg. *Syncarpea*; 25 SSR were found in *C. hongkongensis* subsp. *gigantea*, *C. capitata*, *C. hongkongensis* subsp. *hongkongensis*, and *C. hongkongensis* subsp. *tonkinensis*; 28 SSR were found in *C. elliptica*, *C. kousa*, *C. hongkongensis* subsp. *elegans*, *C. hongkongensis* subsp. *ferruginea*, and *C. hongkongensis* subsp. *melanotricha*; and 31 SSR were found in *C. multinervosa* (**Figure 6A**). The SSR in protein-coding regions was less different, but not the SSR in spacer and introns. These results suggest that SSR can be used as a molecular marker for related research fields such as genetic diversity, evolutionary studies, and phylogeny.

### 3.6 Phylogenetic analysis

**Figure 7:**
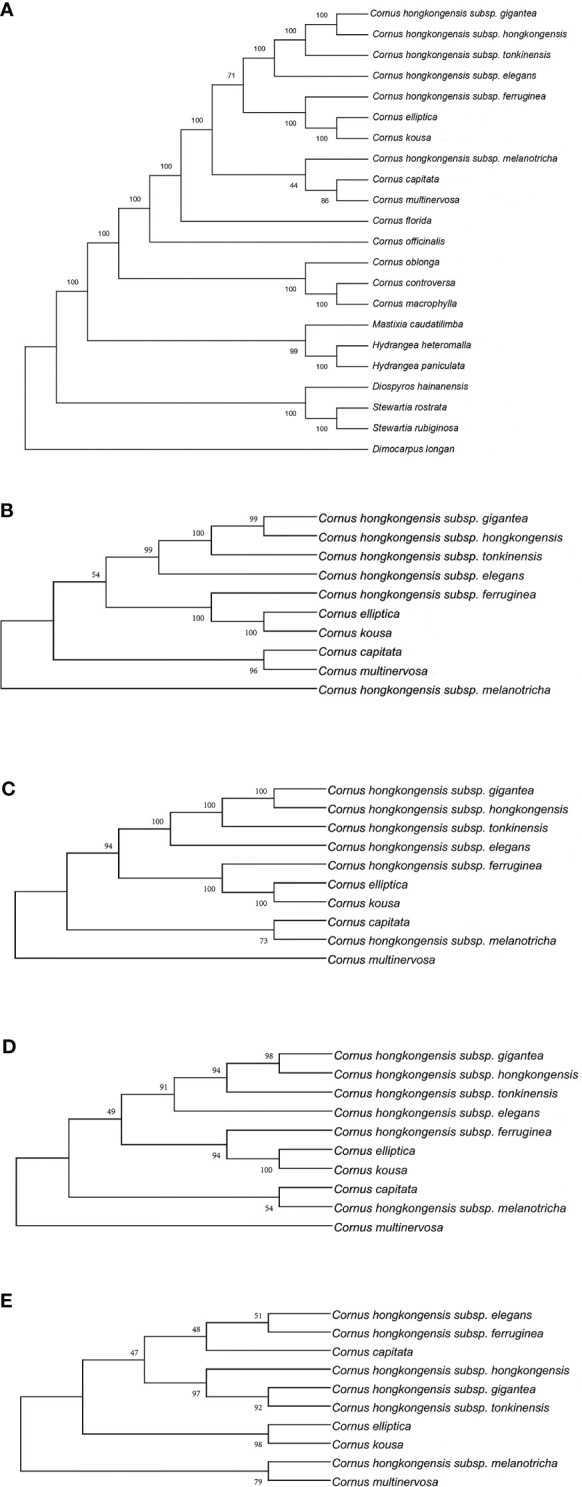
Phylogenetic tree of the chloroplast genome of *Cornus* subg. *Syncarpea* based on the maximum parsimony (MP) analysis. **(A)** Phylogenetic tree constructed using 22 chloroplast genome data. **(B)** Phylogenetic tree constructed using coding region data. **(C)** Phylogenetic tree constructed using LSC region. **(D)** Phylogenetic tree constructed using intron data. **(E)** Phylogenetic tree constructed using IR region. LSC, large single copy; IR, inverted repeat.

**Figure 8:**
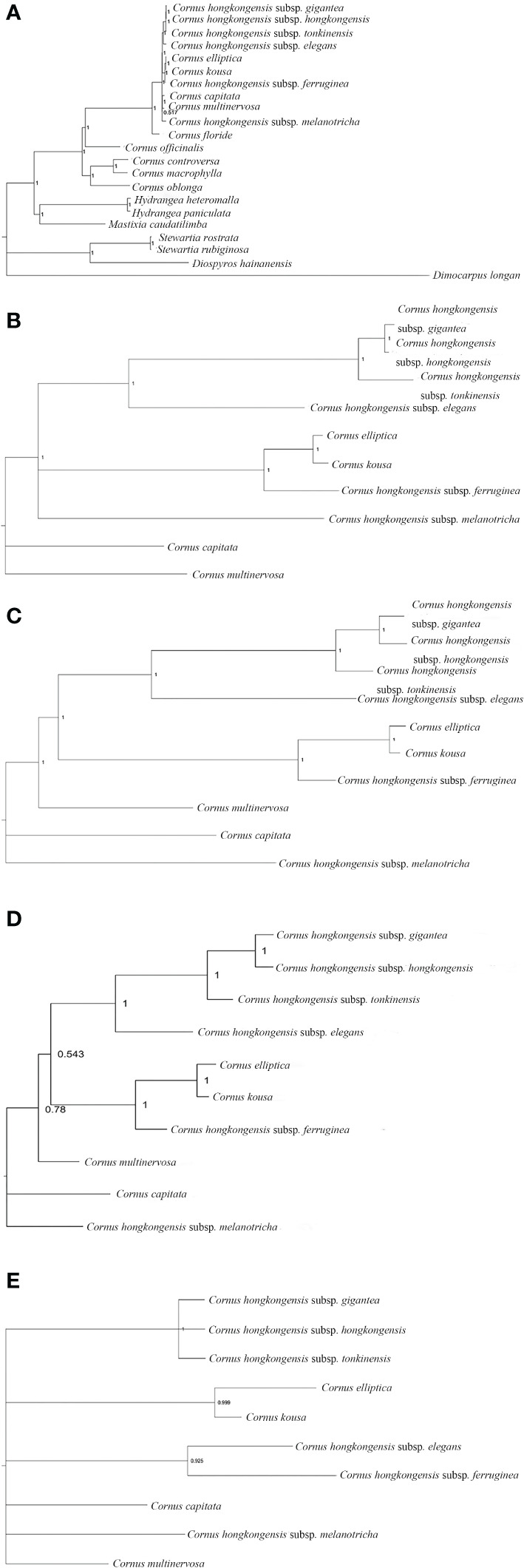
Phylogenetic tree of the chloroplast genome of *Cornus* subg. *Syncarpea* based on the BI analysis. **(A)** Phylogenetic tree constructed using 22 chloroplast genome data. **(B)** Phylogenetic tree constructed using coding region data. **(C)** Phylogenetic tree constructed using LSC region. **(D)** Phylogenetic tree constructed using intron data. **(E)** Phylogenetic tree constructed using IR region. BI, Bayesian inference; LSC, large single copy; IR, inverted repeat.

In this research, we present, for the first time, the cp genome sequences of 10 species and subspecies belonging to the *Cornus* subg. *Syncarpea* and submitted to GenBank; their accession numbers can be found in S3. Then, we constructed a phylogenetic tree using PE and BI methods based on five data sets (**Figures 7**, **8**).

Whether PE or BI trees, we observed that two Saxifragaceae species, namely, *Hydrangea paniculata* and *Hydrangea heteromalla*, are closely related to *Mastixia caudatilimba* of Cornaceae, while *M. caudatilimba* exhibits a distant relationship with other Cornaceae species (**Figures 7A**, **8A**).

*C. hongkongensis* subsp. *hongkongensis* formed a sister branch with *C. hongkongensis* subsp. *gigantea*, while *C. elliptica* formed a sister branch with *C. kousa*. Within the results of the phylogenetic tree, it is evident that *C. hongkongensis* subsp. *tonkinensis*, *C. hongkongensis* subsp. *elegans*, *C. hongkongensis* subsp. *ferruginea*, and *C. hongkongensis* subsp. *melanotricha* manifest as distinct branches, fortified by robust node support values (**Figures 7**, **8**).

According to the MP method, the phylogenetic analysis suggests a close relationship between *C. capitata* and either *C. multinervosa* or *C. hongkongensis* subsp. *melanotricha*, as they form sister branches (**Figures 7A–D**). However, in the BI method-based analysis, there is a proclivity to consider *C. capitata* and *C. multinervosa* as separate branches (**Figure 8**).

In the BI method-based analysis, the phylogeny inferred from the IR region data reveals that *C. hongkongensis* subsp. *tonkinensis*, *C. hongkongensis* subsp. *hongkongensis*, and *C. hongkongensis* subsp. *gigantea* formed a closely related sister branch with a posterior probability of 1. Furthermore, *C. hongkongensis* subsp. *elegans* and *C. hongkongensis* subsp. *ferruginea* also emerged as sister branches with high node support values (PP = 0.925) (**Figure 8E**).

In summary, our analysis of constructing five phylogenetic trees has shed light on the relationship between various subspecies of *C. hongkongensis*. Our findings indicate that *C. hongkongensis* subsp. *hongkongensis* and *C. hongkongensis* subsp. *gigantea* are closely related, leading to the possibility that the latter may be a subspecies of the former. However, it is worth noting that *C. hongkongensis* subsp. *ferruginea* and *C. hongkongensis* subsp. *melanotricha* did not cluster with *C. hongkongensis* subsp. *hongkongensis*, thus suggesting that they should not be classified under the same species.

Furthermore, our results demonstrate an incredibly close relationship between *C. elliptica* and *C. kousa*, pointing toward the possibility that these two plants of *Cornus* subg. *Syncarpea* may indeed belong to the same species with high credibility.

## 4 Discussion

The cp genomes of 10 taxa belonging to *Cornus* subg. *Syncarpea* were analyzed in this study, and it was found that the cp genomes of the 10 *Cornus* subg. *Syncarpea* plants displayed striking similarities in their genome structure and length, GC content, and gene count. The complete genome length spanned from 156,965 bp (*C. hongkongensis* subsp. *tonkinensis*) to 157,383 bp (*C. elliptica*), while the LSC region length varied from 86,296 bp (*C. hongkongensis* subsp. *tonkinensis*) to 86,691 bp (*C. elliptica*), and the SSC region length ranged from 18,386 bp (*C. hongkongensis* subsp. *elegans*) to 18,454 bp (*C. multinervosa*). Moreover, the IR region length oscillated between 26,112 bp (*C. capitata*) and 26,143 bp (*C. multinervosa*), as depicted in **Table 1**. The overall GC content hovered at approximately 38.15%, with only negligible dissimilarities observed among the four regions. The above is consistent with the length and structural features of cp genomes found in other higher plants (Jansen et al., 2005; Daniell et al., 2016). Our analysis revealed an uneven distribution of GC content in the cp genomes of these 10 taxa. The IR regions had a higher GC content compared to the two single-copy regions. This discrepancy may be attributed to the presence of eight rRNA genes with high GC content in the IR regions. No significant differences were observed in gene expression among these 10 taxa of *Cornus* subg. *Syncarpea*, as the number of protein-coding genes, tRNA coding genes, and rRNA coding genes was equal. The cp is a vital organelle in higher plants, responsible for crucial life processes such as photosynthesis and self-replication. The genes involved in the self-replication of the cp include those responsible for the large and small subunits of the ribosomes, DNA-dependent RNA polymerases, ribosomal proteins, rRNA genes, and tRNA genes. Additionally, the genes responsible for photosynthesis, such as Photosystem I and Photosystem II, encompass 33 genes in the cp genome.

This study delves into the cp genomes of 10 species and subspecies belonging to *Cornus* subg. *Syncarpea*, revealing a plethora of gene functions crucial to the proper functioning of this essential organelle, as other plants, the cp genomes of 10 taxa of *Cornus* subg. *Syncarpea*, are conserved in gene length, content, and order, and there is no gene loss. Notably, six genes partake in ATP synthesis, and one of these, the *atpF* gene, contains a single intron. The synthesis of cytochrome *b* involves six genes, while 11 genes are responsible for NADH dehydrogenase synthesis, with *ndhA* and *ndhB* each containing one intron. Additionally, Photosystems I and II require five and 15 genes, respectively, to complete their functions. Four genes, including *rpoC1* with one intron, are involved in RNA polymerase synthesis.

A total of 25 genes are associated with ribosomes, 12 of which contribute to the synthesis of proteins in small subunits, and *rps12* is a trans-spliced gene. Nine genes are responsible for the synthesis of proteins in large subunits, with *rpl2* containing a single intron. Furthermore, four genes play a role in ribosomal RNA synthesis. Finally, seven genes are associated with other cp gene functions, with the *clpP* gene containing two introns. Overall, this thorough analysis of the cp genomes in these 10 taxa of *Cornus* subg. *Syncarpea* provides valuable insights into the complex workings of this critical organelle.

Oligonucleotide repeats, a type of repetitive DNA sequence, are highly prevalent in plastid genomes and have been identified as a valuable tool for pinpointing mutational hotspots (Ahmed et al., 2012; Lee et al., 2014; Abdullah et al., 2020a; Abdullah et al., 2020b; Liu Q. et al., 2020). Specifically, SSRs, or microsatellite DNA, are tandem repeat sequences consisting of several tens of nucleotides, with repeat units typically composed of one to six nucleotides. The flanking sequences of each SSR are typically composed of relatively conserved single-copy sequences. Due to their polymorphism, co-dominance, and reliability, SSRs have emerged as a widely used molecular marker technology based on specific primer PCR (Oliveira et al., 2006; Sonah et al., 2011; Gao et al., 2018). Additionally, SSRs are invaluable for detecting genetic diversity and polymorphisms at the population, intraspecific, and cultivar levels, as well as for distinguishing between species (Thiel et al., 2003; Mader et al., 2018). In our study, we detected two distinct types of SSRs in the cp genomes of 10 species and subspecies of *Cornus* subg. *Syncarpea*, which were primarily located in the LSC region. Among these SSRs, mononucleotide repeats were the most frequently observed. We also found that the number of SSRs in intergenic regions and introns varies greatly, which can be used as potential molecular markers for genetic diversity, evolution, and phylogenetic studies.

Throughout the course of plant evolution, variations in the length of the IR region of the cp genome are a ubiquitous occurrence, leading to the emergence of diverse boundary features (Yang et al., 2010; Wang et al., 2017; Ding et al., 2021). The present study investigated the boundary genes in 10 taxa belonging to *Cornus* subg. *Syncarpea*, identifying *rpl22*, *rps19*, *rps2*, *ycf1*, *ndhF*, *trnH*, and *psbA* as the predominant genes. Overall, these 10 taxa exhibited a high degree of similarity in their boundary features, the gene information is stable during the transcription process, and there are no more obvious signs of recombination, providing evidence for their relative stability among closely related species. However, a few discrepancies were observed in *C. capitata*, *C. kousa*, *C. hongkongensis* subsp. *elegans*, and *C. elliptica*. Notably, the *ndhF* gene in *C. elliptica* (2,244 bp) was slightly longer than in the other species (2,238 bp), while *ycf1* in *C. hongkongensis* subsp. *elegans* (5,571 bp) and *C. capitata* (5,580 bp) was slightly shorter than that in the other species.

The evolution of land plants has seen greater potential for variation in the two single-copy regions than in the IR regions, as revealed by m-VISTA analysis. These findings align with previous studies in other plant taxa (Gu et al., 2016; Xu et al., 2017; Alzahrani et al., 2020), attesting to the significance of these regions in plant evolution. While the protein-coding regions were relatively conserved, variations were also observed in the *atpA-atpF*, *atpB*, and *psaI-ycf4* genes. The high GC content observed in the genome may contribute to the lower variation in tRNA sequences and IR regions, underscoring the significance of GC content in maintaining sequence stability, as previously reported (Necsulea and Lobry, 2007; Kim et al., 2019).

Codon usage preference plays a crucial role in gene expression and affects protein and mRNA levels in the genome (Zhou et al., 2013; Lyu et al., 2020). As shown in the results, codons ending in A/U have RSCU values greater than 1, while those ending in C/G are less than 1. This pattern is consistent across other plants (Wang et al., 2018; Liu X. Y. et al., 2020) and holds true in the present study, as observed in the *Cornus* subg. *Syncarpea* genome. These findings provide further insight into the role of codon usage preference in shaping the genome, ultimately contributing to our understanding of plant evolution.

In the past, the classification of *Cornus* subg. *Syncarpea* was based on morphological data, which resulted in the identification of 4–14 distinct species (Xiang, 1987; Xiang et al., 2005), while with the integration of molecular phylogeny and morphology analysis, the subgenus has been redefined to include four species and eight subspecies (Xiang and David, 2005). Furthermore, the investigation of nrDNA ITS and cp *matK* has been utilized to explore the phylogenetic relationships within the *Cornus* genus. Nevertheless, subsequent research has uncovered the presence of incomplete concerted evolution of ITS within individuals of *Syncarpea* (Hu et al., 2013). In this investigation, we obtained their cp genome sequence for the first time and constructed a phylogenetic tree based on these data to explore the genetic relationships among the species. The results of our phylogenetic analysis strongly support the classification of the “Flora of China”, which suggests that *C. hongkongensis* subsp. *gigantea* is closely related to *C. hongkongensis* subsp. *hongkongensis*, although in the phylogenetic tree constructed by the IR data set of the BI method, *C. hongkongensis* subsp. *hongkongensis* and they cluster together to have a high node support value, and *C. hongkongensis* subsp. *hongkongensis* should be separated in the phylogenetic tree of the other four data sets. Furthermore, our data revealed that *C. hongkongensis* subsp. *ferruginea* was distantly related to both *C. hongkongensis* subsp. *melanotricha* and the other subspecies of *C. hongkongensis*, forming a separate branch, and *C. elliptica* and *C. kousa*, forming another branch. This finding challenges the earlier classification in the “Flora of China” and provides a novel perspective on the relationships between species within *Cornus* subg. *Syncarpea*. Additionally, we note that *C. capitata* is special: in the phylogenetic tree constructed with different data sets, it can be clustered with *C. hongkongensis* subsp. *melanotricha*, *C. multinervosa*, or alone, and its support rate varies from high to low, requiring further research.

Overall, our investigation does not support the classification in the “Flora of China” that *C. hongkongensis* subsp. *elegans*, *C. hongkongensis* subsp. *ferruginea*, *C. hongkongensis* subsp. *gigantea*, *C. hongkongensis* subsp. *melanotricha*, *C. hongkongensis*, and *C. hongkongensis* subsp. *tonkinensis* are subspecies of *C. hongkongensis*. Our investigation yields additional corroboration that *C. hongkongensis* subsp. *tonkinensis*, *C. hongkongensis* subsp. *elegans*, *C. hongkongensis* subsp. *ferruginea*, and *C. hongkongensis* subsp. *melanotricha* distinctly form separate branches. Simultaneously, considering the nodal support values derived from the MP and BI trees, our results substantiate the proposition that *C. capitata* and *C. multinervosa* individually constitute their own unique branches. This work not only adds to our understanding of the genetic relationships among the species of *Cornus* but also provides valuable cp genome information for future research into the origin and differentiation of this group at the cp genome level. It is important to note, however, that our study only analyzed the cp genome of *Cornus* subg. *Syncarpea* plants. Therefore, our results should be confirmed by analyzing different regions to ensure their robustness.

## Data availability statement

The datasets presented in this study can be found in online repositories. The names of the repository/repositories and accession number(s) can be found in the article/**Supplementary Material**.

## Author contributions

BG: Writing – review & editing, Conceptualization, Funding acquisition, Project administration, Resources, Supervision. JW: Writing – original draft, Data curation, Formal analysis, Investigation, Software, Visualization. HG: Investigation, Software, Visualization, Writing – original draft. YL: Conceptualization, Project administration, Supervision, Writing – review & editing.
