# Supplementary material for: Comparative and phylogenetic analyses based on the complete chloroplast genome of Cornus subg. Syncarpea (Cornaceae) species
Source: Front Plant Sci. 2024 Mar 13;15:1306196. doi: 10.3389/fpls.2024.1306196 (PMC10965615; doi:10.3389/fpls.2024.1306196)
Supplement: Supplementary file 1 [file Table_1.docx]

**Supporting information -Table**

**Table S1** Collection location of 10 samples

| Sample | *C. hongkongensis*  subsp*. ferruginea* | *C. hongkongensis*  subsp*. gigantea* | *C. multinervosa* | *C. hongkongensis*  subsp*. melanotricha* | *C.elliptica* |
| --- | --- | --- | --- | --- | --- |
| Collection  Location | Guizhou Forest Park Lushan Mountain | Jiu longjiang  Forest Park | Emei  Mountain | Jinyun Mountain  Emei Mountain | Jinggang  Mountain |
| Sample | *C. capitata* | *C. hongkongensis*  subsp*. elegans* | *C. hongkongensi* | *C. kousa* | *C.hongkongensis*  subsp*. tonkinensis* |
| Collection  Location | Changchong  Mountain | Zhejiang University  Yuquan Campus | Ganzhou  xiakeng | Guizhou  Forest Park | Guizhou  Forest Park |

**Table S2** Data output list

| Sample | Library | Raw Reads | Clean Reads |
| --- | --- | --- | --- |
| *C. hongkongensis* subsp. *gigantea* | FDSW202333541-2r | 13,510,149 | 13,438,753 |
| *C. hongkongensis* | FDSW202333542-2r | 12,523,414 | 12,451,564 |
| *C. multinervosa* | FDSW202333550-2r | 14,104,238 | 14,023,402 |
| *C. hongkongensis* subsp*. elegan* | FDSW202333543-2r | 13,537,264 | 13,461,840 |
| *C. hongkongensis* subsp*. tonkinensis* | FDSW202333544-2r | 13,932,872 | 13,857,456 |
| *C. capitata* | FDSW202333549-2r | 13,339,638 | 13,262,855 |
| *C. hongkongensis* subsp*. melanotricha* | FDSW202333545-2r | 12,840,365 | 12,766,737 |
| *C. hongkongensis* subsp*. ferruginea* | FDSW202333546-2r | 12,952,225 | 23,887,638 |
| *C. kousa* | FDSW202333547-2r | 14,894,202 | 14,814,440 |
| *C. elliptica* | FDSW202333548-2r | 13,876,968 | 13,802,943 |

Sample: name of sample. Raw Reads:The number of Raw Reads were sequenced.

Clean Reads: The number of Clean Reads obtained after filtering.

**Table S3** Accession numbers of 22 species used to construct phylogenetic trees

| Species | Gene database No. / NCBI No. |
| --- | --- |
| *C. florida*  *C. oblonga*  *C. officinalis*  *C. macrophylla*  *C. controversa*  *Mastixia caudatilimba*  *Hydrangea paniculata*  *Hydrangea heteromalla*  *Stewartia rostrata*  *Stewartia rubiginosa*  *Diospyros hainanensis*  *Dimocarpus longan*  *C. multinervosa*  *C. capitata*  *C. elliptica*  *C. kousa*  *C. hongkongensis* subsp*. ferruginea*  *C. hongkongensis* subsp*. melanotricha*  *C. hongkongensis* subsp*. tonkinensis*  *C. hongkongensis* subsp*. elegans*  *C. hongkongensis* subsp*. gigantea*  *C. hongkongensis* | NC_044820.1  NC_044811.1  MH729079.1  NC_044810.1  KU852492.1  MG525001.1  NC_044829.1  NC_044842.1  KY406789.1  MH753080.1  NC_042160.1  NC_037447.1  OR597575  OR597576  OR597577  OR597578  OR597579  OR597580  OR597581  OR597582  OR597583  OR597584 |
